# Farmed blue mussels (*Mytilus edulis*)—a nutrient-dense resource retaining nutritional value through processing

**DOI:** 10.3389/fnut.2024.1443229

**Published:** 2024-11-01

**Authors:** Hanne Bjerknes, Edel O. Elvevoll, Monica Alterskjær Sundset, Andreas Langdal, Karl-Erik Eilertsen

**Affiliations:** ^1^Norwegian College of Fishery Science, UiT The Arctic University of Norway, Tromsø, Norway; ^2^Department of Arctic and Marine Biology, UiT The Arctic University of Norway, Tromsø, Norway

**Keywords:** biochemical composition, LC-PUFAs, amino acids, taurine, aquaculture, environmental footprints, nutrition

## Abstract

This study investigated how farmed blue mussels (*Mytilus edulis*) can optimize human nutrient intake. A particular focus was on assessing nutrient preservation during steaming and freeze-drying, processes that could deplete nutrients. The study compared the content of essential amino acids and fatty acids in steamed and freeze-dried blue mussels to the nutritional needs of humans and farmed Atlantic salmon (*Salmo salar*). Additionally, it assessed the ethyl acetate method versus the traditional, more toxic Folch method for lipid extraction from blue mussels. Both steaming and freeze-drying effectively preserved essential amino acids and fatty acids in blue mussels. A 100 g serving of steamed blue mussels contributes from 26.8 ± 0.78% (Phe) to 54.9 ± 1.66% (Thr) of the daily recommended intake of essential amino acids (EAA). For steamed freeze-dried blue mussels, over 100% of the recommended intake is met for all EAA and as much as 243% for threonine. The 100 g serving will also provide 271 mg eicosapentaenoic acid (EPA; 20:5n-3) and 220 mg docosahexaenoic acid (DHA; 22:6n-3), thus covering the required intake of n-3 long-chain polyunsaturated fatty acids for adults as well as the recommended intake for pregnant and lactating women. Mussels are non-fed filter feeders that generally provide these nutrients with significantly lower environmental footprints, measured as global warming, eutrophication, and acidification, compared to farmed Atlantic salmon. Blue mussels can also be a valuable feed ingredient for farmed Atlantic salmon. Finally, it was demonstrated that the ethyl acetate method is not suited for lipid extraction from blue mussels, as the lipid yield was only half compared to the lipid yield using the Folch method.

## 1 Introduction

The rapidly growing global population demands increased food production and supply of nutrients to ensure food security and prevent malnutrition (1). The ocean may contribute to improved food security and nutrition from underexploited resources, such as lower trophic marine food sources (2, 3). In recent decades, we have observed the consequences of consumption of foods high in energy but low in nutrient density, as the global epidemic of overweight and obesity is a challenge all over the industrialized world accompanied by numerous lifestyle diseases (4). Food production is central to human health and the environment, and there is an urgent need to produce nutritious foods with low climate impact (5–7). Seafood provides a nutrient-dense source with favorable amino acid composition and numerous minerals. It is also a unique source of n-3 long-chain polyunsaturated fatty acids (n-3 LC-PUFA) with proven health benefits, such as reducing the risk of cardiovascular diseases (8, 9).

Blue mussels (*Mytilus edulis*) are an environmentally sustainable (10), low-trophic resource that can be further exploited as food for humans and aquaculture feed. As filter feeders, they rely on plankton organisms without needing to be fed fishmeal or fish oil, resources that often face substantial sustainability issues (11).

Blue mussels are typically steamed before eaten. This heat treatment has several purposes, including reducing the risk of foodborne illnesses and enhancing palatability (12–14). However, steaming or boiling seafood can deplete nutrients from the raw materials, particularly water-soluble nutrients like the beneficial, non-proteinogenic amino acid taurine, which is substantially reduced during boiling of seafood such as cod, oysters, and shrimp (15, 16).

Freeze-drying is a method used for the extended or long-term preservation of food items (17). Blue mussels provide all essential amino acids (EAA), are a great source of n-3 LC-PUFA, and contain vitamins and minerals such as B12, selenium, iron, and zinc (18). However, more information is needed on how much of these nutrients are preserved or lost during steaming and freeze-drying.

Norway is a leading producer of farmed fish, particularly Atlantic salmon (*Salmo salar*). The shift in farmed salmon feed from marine proteins and lipids to more vegetable ingredients has reduced the proportion of n-3 LC-PUFA, while total lipids, especially omega-6 PUFAs, have increased. These changes are also evident in the composition of the salmon muscle (19–23). Although current salmon feed predominantly comprises vegetable sources like soy, incorporating a marine diet with ingredients based on blue mussels may better meet the nutritional needs of salmon and simultaneously increase n-3 LC-PUFA content in the fish fillets (21, 24). In the context of aquaculture production in Norway, blue mussels are currently produced in limited volumes. However, there is substantial potential to increase production as blue mussels can be cultivated and harvested along the Norwegian coast. Low trophic aquaculture has not been a strong tradition in Norway. Given the abundant low-trophic resources, the development of this industry needs to catch up with the salmon farming industry. The blue mussel industry in Norway consists mainly of small companies targeting local markets, with only a few larger companies producing for a broader Scandinavian or European market. The industry has been developing new ways to keep products fresh and delivered on time. One of the new ideas has been to find new uses for by-products, such as small and crushed mussels, to increase their income. Recently, there has been an interest in cultivation in a one-year cycle for use in salmon feed, which has provided new opportunities (25, 26). Blue mussel silage or powder is a promising new marine resource for fish feed (27). However, much development is still needed throughout the entire value chain to ensure a more extensive production of blue mussels. This could help meet the growing demand for marine fatty acids and proteins for direct human consumption, and serve as an alternative feed resource, replacing vegetable sources in salmon feed. Ultimately, this may lead to increased availability of marine nutrients, improved health and welfare, and a reduced environmental footprint of this industry.

The Folch method for lipid extraction has been widely used as the golden standard for lipid extraction of tissues (28). This method uses chloroform and methanol to isolate lipids efficiently. However, due to the hazardous nature of these chemicals, there is a need to develop safer and more environmentally friendly lipid extraction methods involving less toxic solvents. As a nation deeply rooted in fisheries and aquaculture, the Norwegian standardization organization “Standard Norge” established standards early on documenting the quality of fish and seafood products. Ethyl acetate (EtOAc) is commonly used as a solvent for lipid extraction when evaluating salmon quality (NS9402). This method is more straightforward, less time-consuming, and uses less hazardous solvents than the Folch method. It may also be better suited for lipid extraction from sources other than salmon.

This study aimed to investigate whether the content of nutrients such as amino acids and fatty acids in blue mussels are kept intact after household preparation through steaming. We also sought to compare the central nutrients in blue mussels with alternative marine food sources from an environmental sustainability perspective. Additionally, the study explored whether freeze-drying preserved the nutritional integrity of the mussels and assessed the content of umami-flavoured amino acids, which enhance the palatability of food. Finally, the study compared the efficiency of the less toxic, environmentally friendly solvent EtOAc with the gold standard Folch method for lipid extraction.

## 2 Materials and methods

### 2.1 Standards and chemicals

Norleucine (TLC), the amino acid standards A6407 and A6282 and the fatty acid methyl ester standards PUFA1, PUFA2 and PUFA3 were purchased from Supelco Ltd. (Bellefonte, PA, USA). The free fatty acid standard GLC-502 was obtained from Nu-Chek-Prep, Inc. (St. Elysian, MN, USA). Hydrochloric acid (37%, AnalaR grade), dichloromethane (AnalaR), sodium chloride (NaCl) (ACS), ethyl acetate (AnalaR), n-heptane (AnalaR), sulfuric acid (H_2_SO_4_) (ACS reagent) and methanol (AnalaR) were obtained from VWR International (Darmstadt, Germany). Heptadecanoic acid (C17:0), sodium sulfate (Na_2_SO_4_) and 5-sulfosalicylic acid dihydrate (>98%, ReagentPlus) were obtained from Sigma-Aldrich, (St.-Louis, MO, USA). Lithium loading buffer was purchased from Biochrom Ltd. (Cambridge, UK).

### 2.2 Raw material

Blue mussels (*M. edulis*) were harvested on January 23, 2023, from Årsand in Bindal, Trøndelag, Norway, provided by Snadder & Snaskum AS. Raw blue mussels were cut open with a knife, and their contents were scraped out and collected with the remaining liquid within the shells. Once 2 kg of this mixture was collected, it was immediately frozen at −22°C. The steamed blue mussels were prepared by steaming intact raw blue mussels for 6 min. Only the mussels that opened during steaming were retained. The contents were scraped out of their shells and collected with the liquid associated with the tissue. Once 2 kg of this mixture was gathered, it was immediately frozen at −22°C. The frozen raw and steamed blue mussel mixtures were further homogenized before analysis using a Retsch GM300 grinder to eliminate the variation between individual mussels. The grinding container was kept cool using dry ice, and the homogenates were kept at −22°C after homogenization. Half of the homogenate of steamed blue mussels was subsequently freeze-dried to obtain the steamed freeze-dried blue mussel group (Scanvac CoolSafe, LaboGene AS, Denmark). For the different analyses conducted in this study, samples were taken from these three homogenates: raw, steamed, and steamed freeze-dried blue mussels.

### 2.3 Energy

The energy content was measured using a 6725 semi-micro calorimeter with a 6772 calorimetric thermometer and a 1109A oxygen combustion vessel (Parr Instrument Company, Moline, Illinois, US). The combustion vessel was fitted with a 10 cm NiCr fuse wire, and the dewar bucket was filled with 450 g of water. Freeze-dried raw and steamed blue mussels were measured to 40 mg and placed in the combustion vessel under 30 atm of 99.5% oxygen (Linde Gas AS, Oslo, Norway). The combustion vessel was placed in the dewar bucket, where water was continuously stirred. Once the calorimetric thermometer attained a measuring equilibrium in the water temperature, the vessel was ignited. The temperature rose in the combustion vessel, and the dewar bucket was measured every 10 s. Once the combustion was finished, the oxygen in the vessel was removed, and the remaining fuse wire was removed and measured. The caloric value (2.3 cal/g/cm) of the remaining wire was subtracted from the analysis. The total energy content of the sample was retrieved from the calorimetric thermometer according to protocol (29).

### 2.4 Water and ash

Water contents were determined following the AOAC 950.46B method (30). For this, 10 grams of homogenized raw and steamed material and 2 grams of steamed freeze-dried material were weighed and subsequently dried on aluminum beakers in a drying oven at 105°C until a constant weight was achieved. Ash contents were determined by subsequent combustion at 540°C for 16 h according to the AOAC 938.08 method.

### 2.5 Total amino acids

In a tube, 200 mg of raw or steamed blue mussels were mixed with 0.5 ml deionized Milli-Q H_2_O (mH_2_O). For the steamed freeze-dried blue mussel group, 40 mg material was added 0.7 ml mH_2_O. After adding 0.5 ml 20 mM norleucine and 1.2 ml concentrated hydrochloric acid, all samples were flushed with N_2_-gas and put in a heat block (Drybath Stdrd, Thermo Scientific, China) at 110°C for 24 h. Cooled samples were centrifugated at 18,400 × *g* for 5 min, and 1 ml was transferred to new tubes and evaporated to dryness under a steam of N_2_-gas. Finally, the samples were diluted with lithium loading buffer (pH 2.2) and subjected to total amino acid (TAA) analysis. This was performed using a Biochrom 30^+^ amino acid analyzer (Biochrom, Co, Cambridge, UK) as described previously by Mæhre et al. (31). The sum of the molecular weights of each amino acid residue with the subtraction of water mass was used to calculate the protein contents.

Dietary recommendations by FAO/WHO (32) of an adult weighing 70 kg were used to compare the contents and assess to what degree of EAA in steamed and steamed freeze-dried blue mussels fulfill the requirements. The first limiting amino acid in the blue mussels was identified (phenylalanine) and used to calculate the minimum required amount that must be ingested daily if one’s intake of EAAs is to be met solely through blue mussel consumption. It was also calculated how much of the EAAs a 100 g serving of blue mussels could provide.

### 2.6 Free amino acids

In a tube, 1 mg of raw or steamed, or 200 mg of steamed freeze-dried blue mussels, were added 1 ml 20 mM norleucine and 9 ml mH_2_O. After homogenization using an UltraTurrax homogenizer (IKA Werke GmbH, Staufen, Germany), 1 ml 35% 5-sulfosalicylic acid dihydrate (SSA) (Sigma-Aldrich) was added and samples centrifuged at 4,000 × *g* for 10 min. One ml of the supernatant was centrifuged again at 13,500 g for 5 min. Finally, 200 μl of the supernatant was mixed with 800 μl loading buffer and submitted to amino acid analysis for TAA as described by Mæhre et al. (31).

### 2.7 Lipid contents

Total lipid contents were analyzed using two different methods. Each sample was processed in six replicates from the homogenate of raw, steamed, and steamed freeze-dried material.

Lipid extraction was performed using the Folch method (28), with some modifications as described. For the raw and steamed blue mussels, 0.5 g of homogenized sample material was weighed into tubes. Of steamed freeze-dried blue mussels, 0.1077 g was added (corresponds to the dry weight of 0.5 g wet material). The steamed freeze-dried blue mussels were also rehydrated with distilled water until 0.5 g was reached. Then, 10.0 ml dichloromethane:methanol (DCM:MeOH, 2:1) was added to each tube and mixed with an UltraTurrax homogenizer (IKA Werke GmbH, Staufen, Germany) until the materials were completely dissolved. The tubes were subsequently shaken for 25 min on a shaker (Heidolph Multi reax, Schwabach, Germany). After, the tubes were centrifuged at 15,000 × *g* for 10 min at 20°C. The resulting liquid phase was decanted into new centrifuge tubes and added 2.1 ml of a 0.9% NaCl solution. The tubes were gently turned a few times and once again centrifuged, this time at 4,500 × *g* for 10 min at 20°C. After centrifugation, the tubes contained two distinct layers: a water/methanol layer at the top and a DCM/lipid layer at the bottom. The layer containing the lipids was carefully transferred to pre-weighted glass tubes. The solvent (DCM) was evaporated under a stream of nitrogen. The tubes were weighed once again, and the fat contents were calculated.

Lipids were also extracted using the ethyl acetate extraction method. 4.0 g material of raw and steamed blue mussels was weighed into tubes. For the steamed freeze-dried blue mussel material, 0.862 g was used. Then, Na_2_SO_4_ was added and stirred until the material was dry. For the raw blue mussels, this required 12 g, the steamed material required 8 g, and the steamed freeze-dried required 2.0 g. 20 mL of ethyl acetate was added to all tubes, and the mixture was homogenized using an UltraTurrax homogenizer. The tubes were then shaken for 60 min on a Heidolph Multi Reax shaker. The solution was filtered (597, Ø150 mm) into new tubes. One 3.0 ml aliquot was pipetted out of the filtrate, transferred to pre-weighed glass tubes, and evaporated to dryness using nitrogen gas. The tubes were weighed once again, and the fat contents were calculated.

### 2.8 Fatty acid composition

For fatty acid determination, lipids were extracted using the Folch and the ethyl acetate methods as described above, except that parts of the extraction solvent were substituted with internal standard, heptadecanoic acid (C17:0), to 10 mg/ml in DCM:MeOH (2:1) or ethyl acetate, depending on the lipid extraction protocol. For both lipid extract methods, the extracted lipids were dissolved in DCM:MeOH (2:1) to a concentration of 10 mg/ml. Then, 100 μl of the samples were added to Duran tubes with 0.9 ml DCM and 2 ml 2% H_2_SO_4_ in methanol and heated at 100°C for 1 h. All samples were then added 3.5 ml heptane and 3.5 ml 5% NaCl and mixed. The upper phase, which consisted of heptane and lipids, was pipetted into new tubes and evaporated to dryness using N_2_ gas. Finally, the samples were dissolved in 100 μl heptane, transferred to GC tubes, and analyzed on a GC-FID (Agilent 6,890 N) as described previously (33).

Values from Ruyter et al. (34) were used to compare the essential fatty acid (EFA) requirements of Atlantic salmon to the contents of steamed freeze-dried blue mussels. For this, EFA values obtained from the Folch extraction were used.

### 2.9 Carbohydrate contents

The carbohydrate contents were calculated as the remaining weight after subtracting water, ash, protein, and lipid contents from the total weight of 100 grams of wet and dry blue mussel homogenates.

### 2.10 Statistical analysis

All statistical analyses and graphs were performed and created using GraphPad Prism (version 10.1.1). Data on energy contents were compared using an unpaired t-test. Data comparing the lipid extraction methods were assessed using a two-way analysis of variance (ANOVA) multiple comparison test when comparing the means, followed by a Tukey *post hoc* test. Proximates, amino acids, and fatty acids were compared using one-way ANOVA, followed by a Tukey *post hoc* test. Statistical significance was tested at a 0.05 probability level.

## 3 Results

### 3.1 Proximate composition of blue mussels

Steamed freeze-dried blue mussels had significantly higher energy contents (455 ± 6.9 kcal/100 g compared to freeze-dried raw mussels (434 ± 19.1 kcal/100 g, *p* = 0.04). When calculated for wet weight, raw and steamed blue mussels comprise 92.3 ± 4.1 kcal/100 g and 97.4 ± 1.5 kcal/100 g, respectively. Raw and steamed blue mussels exhibited similar water contents, with raw at 78.7 g/100 g and steamed at 78.6 g/100 g on a wet weight (WW) basis (Supplementary Table 1). The water within the shells of both the raw and the steamed material was included in the samples, and this might explain why the content did not decrease after steaming. On a WW basis, the raw blue mussels had lower lipid and protein contents but higher ash contents compared to their steamed counterparts. However, when assessed on a dry weight (DW) basis (Table 1), steaming markedly increased the protein contents from 33.8 g/100 g to 43.8 g/100 g and the lipid contents from 9.13 g/100 g to 12.5 g/100 g while reducing the ash contents from 9.79 g/100 g to 7.22 g/100 g. Freeze-drying resulted in a slight reduction in the protein content to 41.3 g/100 g DW and decreased the ash contents to 6.85 g/100 g DW, but had no significant impact on the lipid content.

**Table 1:** Proximate composition of blue mussels.

Sample	Protein	Lipid	Ash	Carbohydrate (Calculated)
Raw (DW)	33.8 ± 1.17^a^	9.13 ± 0.95^a^	9.79 ± 0.16^a^	47.3
Steamed (DW)	43.8 ± 1.13^b^	12.5 ± 0.53^b^	7.22 ± 0.21^b^	36.5
Steamed freeze-dried (DW)	41.3 ± 1.20^c^	12.5 ± 0.18^b^	6.85 ± 0.09^c^	39.3

All results are displayed as g/100 g dry weight (DW). Values are mean ± standard deviation of *n* = 5 for water and ash content and *n* = 6 for protein and lipids (Folch extraction). Within each column, statistical differences between processing groups are denoted with different letters.

### 3.2 Total and free amino acids

When total amino acids were analyzed, all EAAs were detected, with lysine being the most abundant (47.5 ± 1.26 mg/g DW in steamed mussels), as shown in Table 2. Leucine ranked as the second most prevalent EAA, followed by threonine. Among the non-essential amino acids (NEAA), glutamic acid was predominant (75.2 ± 2.17 mg/g DW in steamed blue mussels), along with arginine (not in raw blue mussels), aspartic acid, and taurine. Generally, steamed and freeze-dried mussels exhibited higher amino acid contents than their raw counterparts, as evidenced by increased total amounts of both EAA and NEAA. The exception is for glycine, hydroxyproline, and taurine, which were most abundant in the raw blue mussels. Across all groups, the EAA to TAA ratio was between 37 and 38%.

**Table 2:** Total amino acid profile in raw, steamed and steamed freeze-dried blue mussel.

	Raw [mg/g (DW)]	Steamed [mg/g (DW)]	Steamed freeze-dried [mg/g (DW)]
Essential amino acids (EAA)			
Histidine	8.84 ± 0.58 ^a^	13.3 ± 0.30 ^b^	12.2 ± 0.51 ^c^
Isoleucine	18.7 ± 0.73 ^a^	23.9 ± 0.67 ^b^	22.4 ± 0.44 ^c^
Leucine	28.2 ± 1.16 ^a^	35.4 ± 0.92 ^b^	33.8 ± 0.68 ^c^
Lysine	36.4 ± 1.75 ^a^	47.5 ± 1.26 ^b^	45.5 ± 1.44 ^b^
Methionine	8.56 ± 0.47 ^a^	11.3 ± 0.46 ^b^	10.9 ± 0.33 ^b^
Phenylalanine	17.6 ± 0.59 ^a^	22.1 ± 0.64 ^b^	21.2 ± 0.49 ^c^
Threonine	22.9 ± 0.71 ^a^	27.17 ± 0.82 ^b^	25.9 ± 0.79 ^c^
Tyrosine	14.7 ± 0.89 ^a^	18.0 ± 1.39 ^b^	17.4 ± 2.32 ^a, b^
Valine	20.2 ± 0.86 ^a^	24.7 ± 0.81 ^b^	23.3 ± 0.77 ^c^
Total EAA	161 ± 6.31 ^a^	205 ± 5.22 ^b^	195 ± 4.66 ^c^
Non-essential amino acids (NEAA)			
Alanine	31.3 ± 1.29 ^a^	34.1 ± 0.79 ^b^	32.1 ± 1.15 ^a^
Arginine	10.1 ± 1.91 ^a^	40.5 ± 2.07 ^b^	35.8 ± 9.27 ^b^
Aspartic acid^*^	31.1 ± 1.11 ^a^	39.9 ± 1.20 ^b^	37.4 ± 1.03 ^c^
Cysteine	3.96 ± 0.22 ^a^	5.43 ± 0.26 ^b^	4.9 ± 0.33 ^c^
Glutamic acid*	61.9 ± 2.24 ^a^	75.2 ± 2.17 ^b^	71.0 ± 2.71 ^c^
Glycine	43.0 ± 1.86 ^a^	42.6 ± 1.03 ^a^	40.2 ± 1.23 ^b^
Hydroxyproline	2.67 ± 0.30 ^a^	2.25 ± 0.51 ^a^	2.4 ± 0.74 ^a^
Proline	20.0 ± 0.60 ^a^	23.5 ± 1.17 ^b^	22.5 ± 0.78 ^b^
Serine	23.7 ± 0.86 ^a^	27.3 ± 0.86 ^b^	26.3 ± 0.80 ^b^
Taurine	34.7 ± 0.82 ^a^	26.5 ± 0.80 ^b^	23.3 ± 1.03 ^c^
Total NEAA	273 ± 10.0 ^a^	335 ± 8.77 ^b^	313 ± 10.9 ^c^
EAA + NEAA	434 ± 14.4 ^a^	540 ± 13.5 ^b^	508 ± 14.4 ^c^

Values are mean ± standard deviation of *n* = 6. Values are shown as mg/g DW. Within the same row, statistical differences between processing groups are denoted by different letters.

* Glutamine and asparagine are included in glutamic acid and aspartic acid, respectively, since the former are deaminated during acid hydrolysis. Tryptophan is destroyed during acid hydrolysis.

The free amino acids (FAA) in blue mussels were primarily composed of NEAA, with nine different NEAA identified (Table 3). Taurine was the most abundant, with glycine and alanine also present in substantial quantities. When analyzing FAA, three EAA were detected with lysine being the most abundant.

**Table 3:** Free amino acid profile of raw, steamed and steamed freeze-dried blue mussel.

	Raw [(mg/g DW)]	Steamed [(mg/g DW)]	Steamed freeze-dried [(mg/g DW)]
Essential amino acids (EAA)			
Histidine	1.20 ± 0.08 ^a^	1.40 ± 0.03 ^b^	1.32 ± 0.07 ^b^
Lysine	3.36 ± 0.19 ^a^	2.87 ± 0.05 ^b^	2.48 ± 0.04 ^c^
Threonine	2.32 ± 0.05 ^a^	1.78 ± 0.10 ^b^	1.75 ± 0.08 ^b^
Total EAA	6.88 ± 0.25 ^a^	6.05 ± 0.12 ^b^	5.54 ± 0.06 ^c^
Non-essential amino acids (NEAA)			
Alanine	11.8 ± 0.37 ^a^	9.04 ± 0.16 ^b^	8.41 ± 0.06 ^c^
Arginine	4.17 ± 0.20 ^a^	2.96 ± 0.21 ^b^	3.43 ± 0.23 ^c^
Aspartic acid	2.50 ± 0.10 ^a^	2.93 ± 0.09 ^b^	2.73 ± 0.08 ^c^
Glutamic acid	6.93 ± 0.32 ^a^	5.36 ± 0.53 ^b^	4.40 ± 0.05 ^c^
Glutamine	1.42 ± 0.14 ^a^	2.32 ± 0.14 ^b^	2.12 ± 0.07 ^c^
Glycine	19.9 ± 0.66 ^a^	15.2 ± 0.31 ^b^	14.8 ± 0.07 ^b^
Proline	3.33 ± 0.30 ^a^	2.78 ± 0.12 ^b^	2.69 ± 0.37 ^b^
Serine	3.14 ± 0.11 ^a^	2.61 ± 0.08 ^b^	2.77 ± 0.04 ^c^
Taurine	32.7 ± 1.15 ^a^	26.5 ± 0.55 ^b^	23.8 ± 0.23 ^c^
Total NEAA	85.9 ± 3.03 ^a^	69.6 ± 1.80 ^b^	65.2 ± 0.40 ^c^
EAA + NEAA	92.8 ± 3.20 ^a^	75.7 ± 1.88 ^b^	70.8 ± 0.42 ^c^

Values are mean ± standard deviation of *n* = 6. Values are shown as mg/g DW. Within the same row, statistical differences between processing groups are signed with different letters.

Lysine, threonine, alanine, glutamic acid, glycine, proline, and taurine decreased after each processing step, i.e., steaming and freeze-drying. For these amino acids, steaming resulted in a more pronounced decrease compared to freeze-drying.

**Figure 1:**
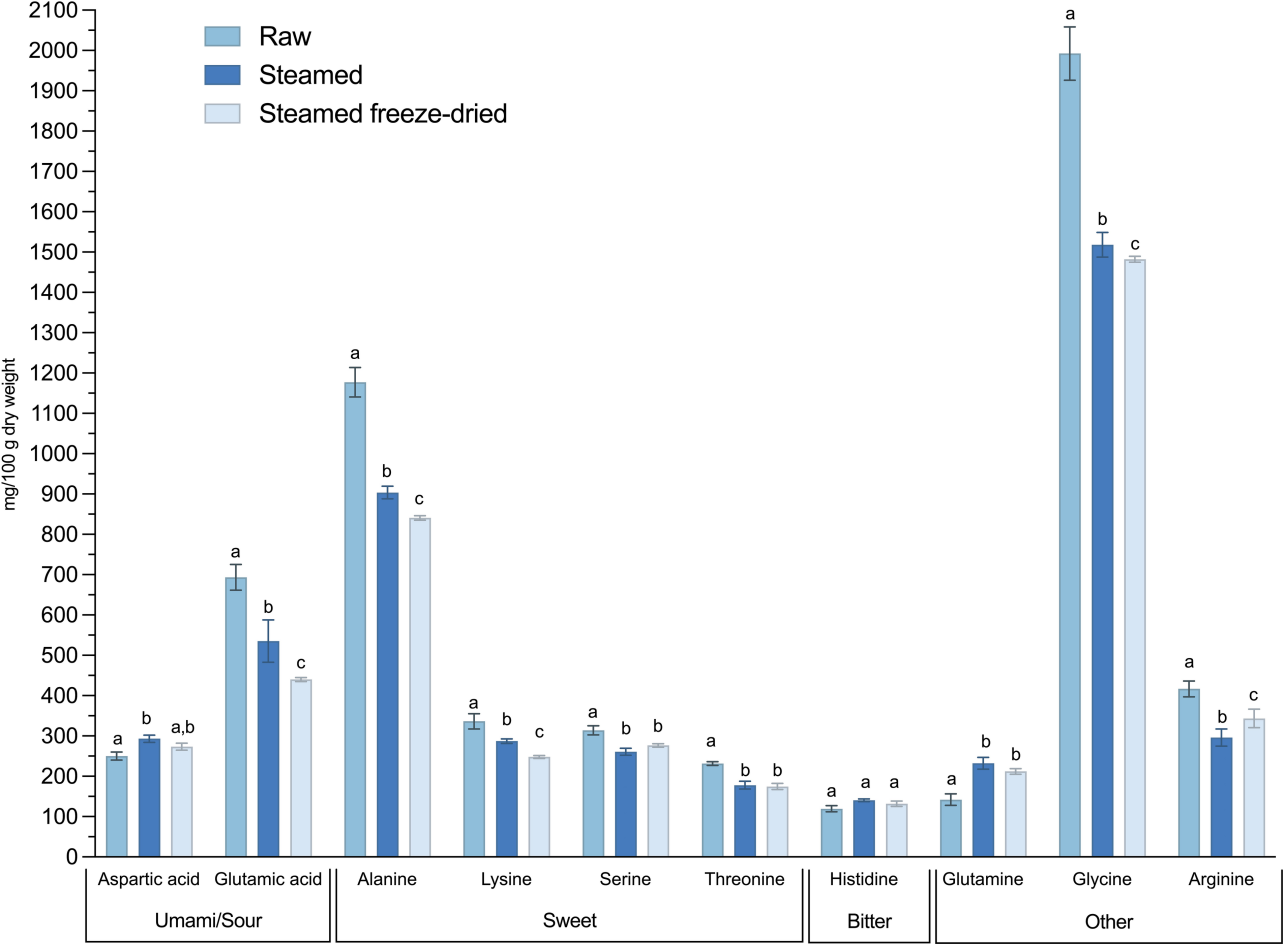
Amino acids (mg/100 DW) of raw, steamed and steamed freeze-dried blue mussels. The data are presented as mean ± standard deviation (SD) of *n* = 6 and the groups were considered as statistically different at *p*-values ≤0.05. Effect of processing was analysed with two-way ANOVA. Differences between processing groups are indicated with small letters according to Tukey’s multiple comparison test.

In Figure 1, the contents of the FAA are arranged according to their respective flavor groups. Although there are statistical differences in the contents before and after steaming or freeze-drying, the levels are well retained. Thus, the taste is expected to be well preserved.

### 3.3 Dietary essential amino acid requirements

**Figure 2:**
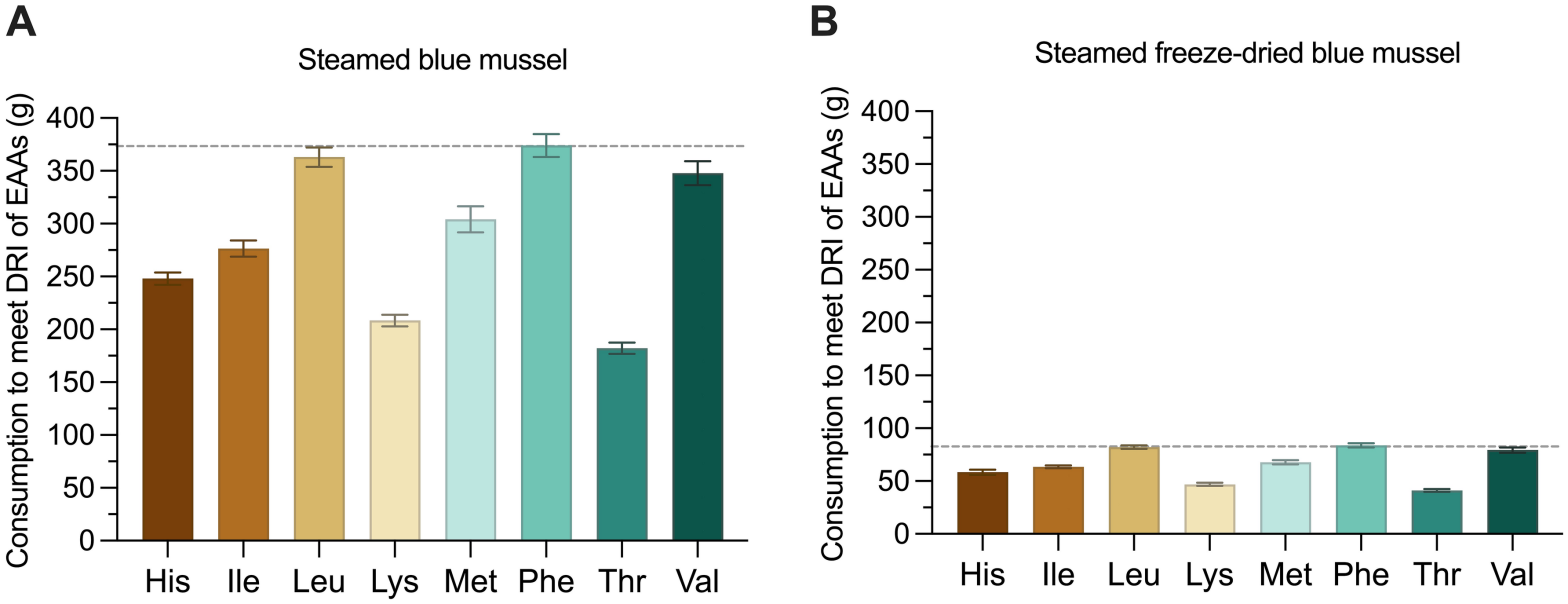
Essential amino acid (EAA) requirement as recommended by FAO/WHO (32) were compared with the EAA content in steamed blue mussel (A) and steamed freeze-dried blue mussel (B). Values are mean ± standard deviation of *n* = 6. The diagram display amount of blue mussels to be ingested to meet the daily requirements for a 70 kg adult of the EAA histidine (His), isoleucine (Ile), leucine (Leu), lysine (Lys), methionine (Met), phenylalanine (Phe), threonine (Thr), and valine (Val). Tryptophan is destroyed during acid hydrolysis and therefore not included. The dashed line represents the required intake of blue mussels, based on the first limiting amino acid phenylalanine.

To meet the daily requirements of EAA, a 70 kg adult needs to consume 374 grams of steamed blue mussels (Figure 2A) or 84 grams of steamed freeze-dried mussels (Figure 2B). This is calculated from the first limiting EAA, which is phenylalanine, followed by leucine and valine for both processing groups. A 100 g serving of steamed blue mussel will contribute with ranges from 26.8% ± 0.78 (Phe) to 54.9% ± 1.66 (Thr) of the daily recommended EAA intake. For steamed freeze-dried blue mussels, over 100% of the recommended EAA intake is met for all EAA and as much as 243% for threonine.

### 3.4 Amino acid contents in blue mussels vs. Atlantic salmon requirements

Values on EAA requirements for Atlantic salmon (*S. salar*) were adapted from Lall and Anderson (35). The contents of EAA (as % of TAA) in blue mussels were compared with the daily requirements of EAA) (See Table 4). When comparing the content of EAA in freeze-dried blue mussels with the feed requirements, all EAA were present in sufficient quantities except for methionine. However, the sum of methionine and cysteine will meet the requirement for sulfur-containing amino acids (1.20 ± 0.05).

**Table 4:** Essential amino acid (EAA) content in steamed freeze-dried blue mussel DW compared to the requirements for salmon.

EAA	EAA requirement of Atlantic salmon, as % of protein	EAA content as % of protein in Steamed freeze-dried blue mussels
Arginine	4.6	8.66 ± 2.18 (188%)
Histidine	1.8, 2.0	2.95 ± 0.06 (163%)
Isoleucine	3.2	5.43 ± 0.13 (169%)
Leucine	5.2	8.19 ± 0.22 (157%)
Lysine	4.1, 3.2–3.6, 6.1	11.02 ± 0.30 (275%)
Methionine	3.1	2.65 ± 0.04 (85.4%)
Phenylalanine + tyrosine	5.8	9.34 ± 0.42 (161%)
Threonine	3.2	6.27 ± 0.15 (196%)
Tryptophan	-	-
Valine	3.9	5.64 ± 0.19 (144%)

The content of each EAA in freeze-dried blue mussles as % of total amino acids were calculated based on the total amino acid profile (Table 2). Values are mean ± standard deviation of *n* = 6. In parentheses, the extent to which each EAA in blue mussels meets the EAA requirement for fish is presented. Values on EAA requirements adapted from “Amino acid nutrition of salmonids: dietary requirements and bioavailability” (35). Tryptophan is not included due to total oxidation during analysis.

### 3.5 Total lipid extraction yields

**Figure 3:**
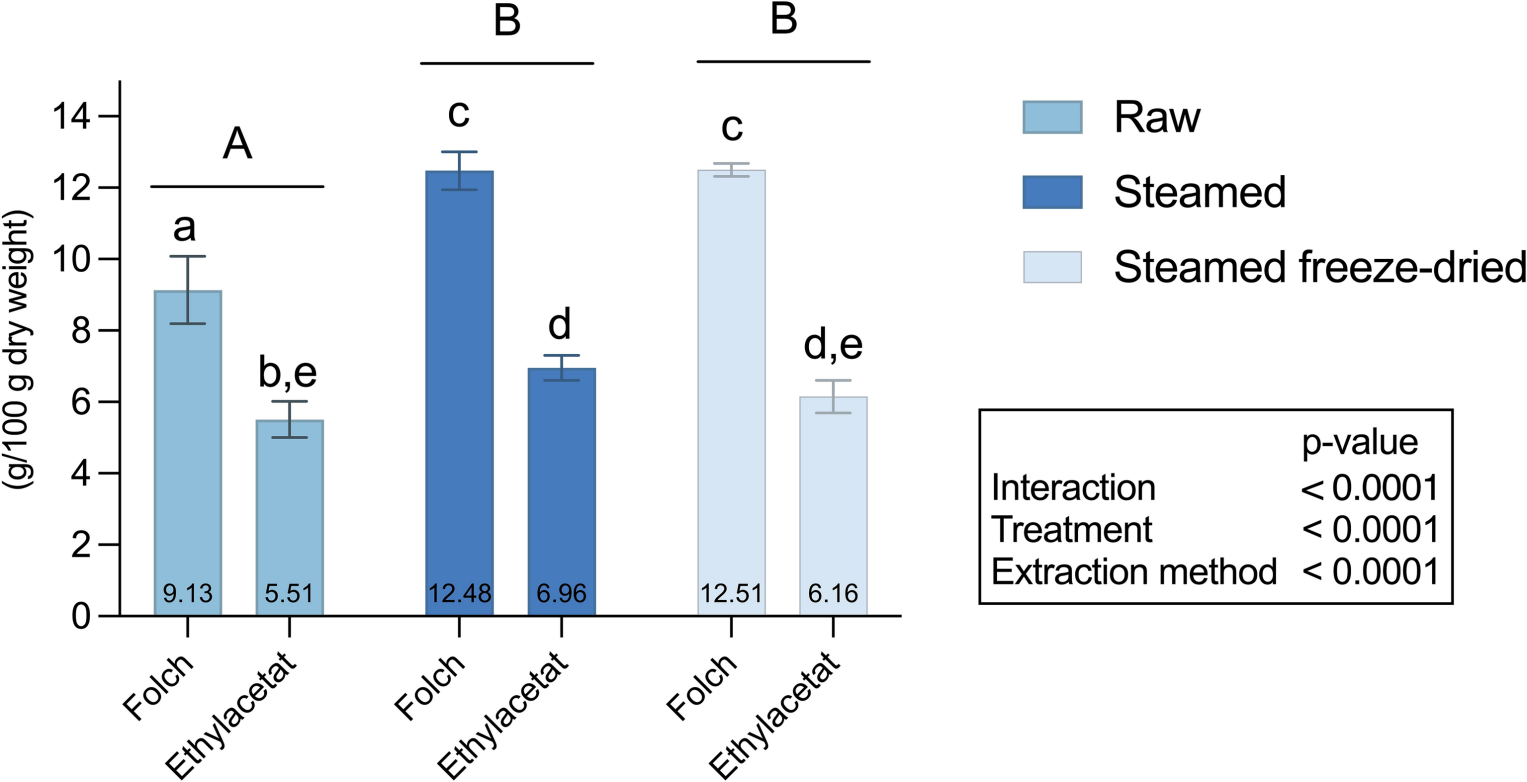
Lipid content (g/100 g DW) of raw, steamed and steamed freeze-dried blue mussel Folch or ethyl acetate extraction (*n* = 6). The data are presented as mean ± standard error (SD) and the groups were considered as statistically different at *p*-values ≤0.05. Annotations of group means are presented at the bottom of each bar. Interaction effect and overall effect from the two factors (processing and extraction method) was analyzed with two-way ANOVA and is presented in the box. Differences between processing groups are indicated with capital letters according to Tukey’s multiple comparison *post hoc* test. Differences between groups (Raw Folch, Raw Ethylacetate, Steamed Folch, Steamed Ethylacetate, Steamed freeze-dried Folch, Steamed freeze-dried Ethylacetate) are indicated with small letters according to Tukey’s multiple comparison test.

Across all processing groups, Folch extraction consistently resulted in a nearly double lipid yield compared to the ethyl acetate extraction from all groups (Figure 3). However, the extraction yield was more than twice as high for the steamed and freeze-dried mussels, 6.16 ± 0.46 versus 12.5 ± 0.18 g/100 g DW mussels. Both extraction methods gave significantly lower lipid yield for raw material than for steamed and steamed freeze-dried mussels (Figure 3).

### 3.6 Fatty acid composition

Overall, palmitic acid (C:16:0), eicosapentaenoic acid (EPA; 20:5n-3), and docosahexaenoic acid (DHA; 22:6n-3) were the three dominating FAs (Table 5). Of the total omega-3 PUFAs present, EPA and DHA comprised 86%, and the n-6/n-3 ratio was low (0.12–0.14), as expected in marine foods. To meet a recommended daily intake ranging from 250 to 500 mg EPA and DHA (36), 51.1 ± 3.4 g to 102 ± 6.7 g of steamed mussels are necessary. Pregnant and lactating women are recommended a DHA intake of minimum 200 mg (37), which may be covered by consuming 91.3 ± 6.0 g of steamed mussels. A serving of 100 grams of steamed blue mussels will provide 271 ± 17.8 mg of EPA and 220 ± 14.4 mg of DHA. This amount covers the required intake of omega-3 PUFAs for adults (491 mg EPA + DHA) and the recommended amount of DHA for pregnant and lactating women.

**Table 5:** Fatty acids [mg FA/g sample dry weight (DW)] of raw blue mussels (R), steamed blue mussels (S), and steamed freeze-dried blue mussels (SFD) from Folch or ethyl acetate extraction.

	Folch extraction			Ethyl acetate extraction		
FA	Raw [(mg/g DW)]	Steamed [(mg/g DW)]	Steamed freeze-dried [(mg/g DW)]	Raw [(mg/g DW)]	Steamed [(mg/g DW)]	Steamed freeze-dried [(mg/g DW)]
14:0	0.88 ± 0.44 ^S, SFD^	1.38 ± 0.09 ^R^	1.42 ± 0.05 ^R, M^	0.88 ± 0.03	1.10 ± 0.01	0.98 ± 0.04 ^M^
16:0	6.82 ± 0.37 ^S, SFD, M^	10.3 ± 0.29 ^R, M^	10.3 ± 0.26 ^R, M^	4.73 ± 0.19 ^S, SFD, M^	5.67 ± 0.07 ^R, SFD, M^	5.16 ± 0.18 ^R, S, M^
16:1n-7	3.78 ± 0.48 ^S, SFD^	6.40 ± 0.34 ^R, M^	5.75 ± 1.11 ^R^	3.79 ± 0.39 ^SFD^	4.24 ± 0.50 ^M^	4.87 ± 0.24 ^R^
18:0	1.46 ± 0.06 ^S, SFD, M^	2.44 ± 0.20 ^R, SFD, M^	2.62 ± 0.07 ^R, S, M^	0.74 ± 0.04 ^M^	0.83 ± 0.02 ^M^	0.85 ± 0.05 ^M^
18:1n-9	0.89 ± 0.11 ^S, SFD^	1.62 ± 0.20 ^R, M^	1.63 ± 0.78 ^R, M^	0.85 ± 0.05	0.96 ± 0.08 ^M^	1.02 ± 0.04 ^M^
18:1n-7	1.21 ± 0.15 ^S, SFD^	1.97 ± 0.15 ^R, M^	1.83 ± 0.26 ^R, M^	1.12 ± 0.08 ^SFD^	1.08 ± 0.10 ^SFD, M^	1.17 ± 0.05 ^S, R, M^
18:2n-6	0.62 ± 0.08 ^S, SFD^	1.01 ± 0.06 ^R, M^	0.98 ± 0.30 ^R, M^	0.58 ± 0.06	0.60 ± 0.07 ^M^	0.63 ± 0.03 ^M^
18:3n-3	0.50 ± 0.08 ^S, SFD^	0.95 ± 0.07 ^R, M^	0.78 ± 0.25 ^R, M^	0.49 ± 0.05	0.51 ± 0.06 ^M^	0.53 ± 0.03 ^M^
20:1 n-15	0.42 ± 0.03 ^S, SFD, M^	0.00 ± 0.00 ^R^	0.00 ± 0.00 ^R^	0.28 ± 0.02 ^S, SFD, M^	0.00 ± 0.00 ^R^	0.00 ± 0.00 ^R^
20:1 n-12^**^	0.79 ± 0.13 ^S, SFD^	2.02 ± 0.11 ^R, SFD, M^	1.76 ± 0.22 ^R, S, M^	0.81 ± 0.07 ^S. SFD^	1.26 ± 0.14 ^R, M^	1.31 ± 0.05 ^R, M^
20:1 n-9	1.17 ± 0.11 ^S, SFD^	1.93 ± 0.12 ^R, M^	1.75 ± 0.24 ^R, M^	0.96 ± 0.09	0.75 ± 0.07 ^M^	0.82 ± 0.04 ^M^
20:2 NMID_1_^*^	0.71 ± 0.07 ^S, SFD^	1.16 ± 0.06 ^R, M^	1.12 ± 0.14 ^R, M^	0.61 ± 0.05	0.66 ± 0.05 ^M^	0.67 ± 0.03 ^M^
20:2 NMID_2_^*^	0.73 ± 0.09 ^S, SFD, M^	1.32 ± 0.13 ^R, SFD, M^	1.00 ± 0.17 ^R, S, M^	0.43 ± 0.04 ^S, M^	0.00 ± 0.00 ^R, SFD, M^	0.42 ± 0.02 ^S, M^
20:2n-6	0.35 ± 0.04 ^S, S FD^	0.55 ± 0.04 ^R, M^	0.47 ± 0.08 ^R, M^	0.31 ± 0.03	0.31 ± 0.03 ^M^	0.31 ± 0.02 ^M^
20:4n-6	0.99 ± 0.11 ^S, SFD, M^	1.75 ± 0.12 ^R, SFD, M^	1.46 ± 0.24 ^R, S, M^	0.74 ± 0.06 ^S, SFD, M^	0.46 ± 0.05 ^R, M^	0.52 ± 0.02 ^R, M^
20:4 n-3^***^	0.13 ± 0.02	0.17 ± 0.42	0.30 ± 0.23	0.12 ± 0.01	0.15 ± 0.09	0.19 ± 0.16
20:5n-3	6.48 ± 0.87 ^S, SFD^	12.7 ± 0.83 ^R, SFD, M^	10.4 ± 1.98 ^R, S, M^	5.63 ± 0.53	5.67 ± 0.72 ^M^	6.05 ± 0.28 ^M^
22:2 NMID_1_^*^	0.97 ± 0.12 ^S, SFD, M^	1.74 ± 0.16 ^R, SFD, M^	1.40 ± 0.25 ^R, S, M^	0.59 ± 0.06 ^M^	0.59 ± 0.08 ^M^	0.66 ± 0.03 ^M^
22:2 NMID_2_^*^	0.18 ± 0.05 ^S, SFD^	0.43 ± 0.02 ^R, SFD, M^	0.29 ± 0.06 ^R, S, M^	0.20 ± 0.02	0.23 ± 0.03 ^M^	0.22 ± 0.01 ^M^
22:5n-3	0.48 ± 0.06 ^S, SFD, M^	0.86 ± 0.05 ^R, SFD, M^	0.73 ± 0.11 ^R, M^	0.37 ± 0.08 ^M^	0.35 ± 0.04 ^M^	0.36 ± 0.02 ^M^
22:6n-3	5.44 ± 0.70 ^S, SFD^	10.3 ± 0.67 ^R, SFD, M^	7.97 ± 1.49 ^R, S, M^	4.97 ± 0.45	4.51 ± 0.58 ^M^	4.46 ± 0.20 ^M^
Sum unidentified	0.74 ± 0.09 ^S,^	1.91 ± 0.69 ^SFD, M^	1.16 ± 0.20 ^R, S^	0.50 ± 0.04 ^S^	1.33 ± 0.20 ^R, M^	0.90 ± 0.03
SFA	9.17 ± 0.82 ^S, SFD, M^	14.1 ± 0.54 ^R, M^	14.3 ± 0.33 ^R, M^	6.34 ± 0.26 ^S, M^	7.59 ± 0.09 ^R, M^	6.98 ± 0.25 ^M^
MUFA	9.18 ± 0.38 ^S, SFD^	13.9 ± 0.86 ^R, M^	13.0 ± 2.11 ^R, M^	7.80 ± 0.68	8.30 ± 0.86 ^M^	9.18 ± 0.38 ^M^
PUFA	17.6 ± 2.26 ^S, SFD^	32.9 ± 2.31 ^R, SFD, M^	26.9 ± 4.92^R, S, M^	15.0 ± 1.37	14.0 ± 1.72 ^M^	15.0 ± 0.76 ^M^
Sum n-6	1.96 ± 0.23 ^S, SFD^	3.30 ± 0.21^R, M^	2.91 ± 0.52 ^R, M^	1.63 ± 0.15	1.38 ± 0.15 ^M^	1.46 ± 0.07 ^M^
Sum n-3	13.0 ± 1.71 ^S, SFD^	24.9 ± 1.79 ^R, SFD, M^	20.2 ± 3.85^R, S, M^	11.6 ± 1.06	11.18 ± 1.42 ^M^	11.6 ± 0.60 ^M^
20:5n-3 + 22:6n-3	11.9 ± 1.56 ^S, SFD^	23.0 ± 1.50 ^R, SFD, M^	18.4 ± 3.47 ^R, S, M^	10.6 ± 0.98	10.2 ± 1.30 ^M^	10.5 ± 0.48 ^M^
Ratio n-6/n-3	0.15 ± 0.00 ^S, M^	0.13 ± 0.00 ^R, SFD^	0.15 ± 0.01 ^S, M^	0.14 ± 0.00 ^S, SFD, M^	0.12 ± 0.00 ^R^	0.13 ± 0.00 ^R, M^
Total	36.7 ± 3.37 ^S, SFD^	66.2 ± 3.99 ^R, SFD, M^	56.8 ± 6.98 ^R, S, M^	30.3 ± 2.14	33.2 ± 2.12 ^M^	33.1 ± 1.22 ^M^

Values are mean ± standard deviation of *n* = 6. Statistical differences within the individual processing groups due to extraction method is denoted with the letter “M.” Within the two extraction methods, statistical differences among the various processing groups R, S, and SFD are denoted by the letter(s) of the processing group from which they are significant different from. SFA saturated fatty acids, MUFA monounsaturated fatty acids, PUFA polyunsaturated fatty acids, n-6 omega-6 fatty acids, n-3 omega-3 fatty acids. FA that constituted more than 0.5% of total fatty acids was included.

* These fatty acids were not identified by our software due to lack of standards; it is assumed that they are correctly identified based on an assumption that we know these fatty acids are present in blue mussels, and the peaks were present in the expected area….

** This could possibly be C18:4 n-3.

*** This could possibly be C22:1 n-11.

In the raw blue mussels, when comparing the yield of FA extracted with Folch or ethyl acetate, only 7 of the 21 identified FA were different using the two methods. However, as many as 18 of the 21 varied when comparing the use of the two methods on steamed and steamed freeze-dried material. Steamed blue mussels had the highest total FA contents using the Folch method, as reflected in total lipid contents (Figure 3).

**Figure 4:**
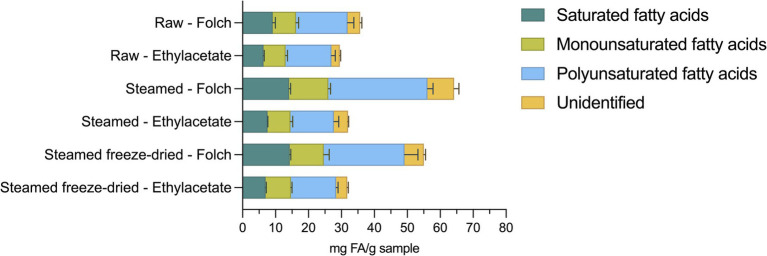
Fatty acid profile of saturated, monounsaturated, polyunsaturated and unidentified fatty acids (mg FA/g sample) from raw blue mussels (R), steamed blue mussels (S), and steamed freeze-dried blue mussels (SFD) extracted with the Folch or ethyl acetate method (*n* = 6). The data are presented as mean ± standard deviation.

The FA profile was generally dominated by PUFAs, which constituted 41.2 to 47.1%, followed by saturated fatty acids (SFA), ranging from 21.5 to 26.0%, and monounsaturated fatty acids (MUFA), ranging from 18.6 to 24.3% (Figure 4).

### 3.7 Availability of essential fatty acids in blue mussels vs. salmon requirements

The dietary requirements of Atlantic salmon were adopted from Ruyter et al. (34), and the contents of the essential fatty acid α-linolenic acid (18:3n-3) and the n-3 LC-PUFAs (EPA, DHA, and DPA) in steamed freeze-dried blue mussels were compared to these (Table 6). The mussels comprised lower amounts of 18:3n-3 than required, while the n-3 LC-PUFAs were present in sufficient quantities.

**Table 6:** Essential Fatty acids (EFA) requirements of Atlantic salmon compared to EFA content in steamed freeze-dried blue mussel from Folch extraction.

EFA of Atlantic salmon (*Salmo salar*)	EFA Requirements of Atlantic salmon (*Salmo salar*; % Dry diet)	EFA content in steamed freeze-dried blue mussels (% Dry diet)
18:3n-3	1.0	0.078 ± 0.03 (7.8%)
n-3 LC-PUFA	0.5–1.0	1.91 ± 0.36 (191–382%)

Reference EFA Requirements of Atlantic Salmon retrieved from (34).

## 4 Discussion

Steamed blue mussels are an excellent food source for those aiming to optimize nutrient intake without consuming to many calories, given their low caloric density (only 97.4 ± 1.48 kcal per 100 grams), and high content of important nutrients. A 100-gram serving contributes significantly to the daily recommended intake of EAA and covers the requirements of EPA and DHA. Although the mineral contents were not investigated in this study, a recent paper provided data on the contribution of blue mussels to the requirements of important vitamins and minerals (18). Hence, compared to foods increasingly consumed today that often are both poor in nutrients and calorie-dense, steamed blue mussels offer a high content of essential nutrients per calorie.

### 4.1 Protein content and impact of preparation

We found that the protein contents in blue mussels increased from 7.18 ± 0.25 g/100 g in raw mussels to 9.29 ± 0.24 g/100 g when steamed for human consumption (Supplementary Table 1). This result is primarily due to water lost during the steaming process, yielding a higher dry matter content. Similarly, cooking has been observed to increase the amino acid content in other types of seafood as well (38). This apparent increase is likely caused by a reduction of non-protein, water-soluble compounds such as minerals and trace elements in addition to free amino acids and carbohydrates. This is supported by the notion that free AA, ash, and carbohydrate decreased after steaming (Supplementary Table 1; Table 3). During steaming, minerals will be carried by the leaking water resulting in a lower ash content in steamed blue mussels as compared to raw blue mussels. The overall biochemical composition of steamed blue mussels in this study is comparable to previously reported data (18, 39). However, Moxness Reksten et al. (18) reported somewhat higher protein levels of 13.6 ± 1.1 g/100 g and 13.2 ± 2.1 g/100 g, respectively, in raw and steamed blue mussels. The difference may be attributed to the different analytical methods used (40). The protein contents in the present study was calculated based on the content of the individual amino acids, according to recommendations by the Food and Agricultural Organization (41). Protein content obtained through total amino acid analysis typically gives lower values than methods used by calculating protein content via a nitrogen-to-protein factor as Moxness Reksten et al. (18). The latter method assumes a constant ratio between nitrogen and protein, which may not be accurate if the organism contains other compounds containing nitrogen, such as ammonia and urea, potentially leading to an overestimation of protein (76). In addition, during the acid hydrolysis necessary for amino acid analysis, tryptophan is destroyed, which also may contribute to a slight underestimation of protein content. Blue mussels contain some tryptophan, although the levels are fairly low (18). Differences in protein content between different studies may also result from seasonal and geographical variations (42).

All the EAA were present in our blue mussels, constituting approximately 38% of the total AA across all groups (Table 2). This underscores the status of blue mussels as an excellent, high-quality protein source. With global demand for marine protein escalating, an increase in the production of blue mussels may contribute to meeting this need. Noteworthy, proteins from marine sources may have additional advantages in the context of obesity prevention over those from terrestrial sources (43–46). This notion was supported by a study reporting that blue mussels reduce weight gain in mice (47). These findings both reinforce the potential of blue mussels as a premier protein source and highlight the possible benefits of marine proteins in combating obesity.

Taurine has been suggested as a conditionally essential functional nutrient since our capacity for synthesis is limited (48). It may enhance the impact of n-3 fatty acids and improve the lipid profile in humans (49). Taurine is not found in plants (except some algae) (50, 51), but it is a good marker for shellfish consumption (52). The contents of taurine, an almost exclusive free amino acid, decreased significantly after steaming and subsequent freeze-drying of the blue mussels (Table 2). This is likely due to its water-soluble nature, which aligns with findings reported by Spitze et al. (16). Although a further reduction in taurine contents was observed after freeze-drying, this reduction was less pronounced. However, steamed blue mussels still contained considerable amounts of taurine, with 26.5 ± 0.55 mg/g (dry weight) compared to 34.7 ± 0.82 mg/g before steaming. Consuming the remaining broth when steaming blue mussels helps recapture the taurine that leaches out during steaming.

The contents of EAA in blue mussels were found to be well preserved during the steaming process. A meal of blue mussels for an adult typically includes about 200 grams of mussel meat (53). If blue mussels were to meet the EAA requirements, a consumption of as much as 374 grams daily of steamed blue mussels would be required, according to the findings presented in this study (Figure 2A). The first limiting amino acid is phenylalanine, followed by leucine and valine. Blue mussels also contain aspartic and glutamic acid in significant amounts (Figure 1). These amino acids enhance the umami taste often associated with meat. Umami plays a crucial role in enhancing flavor, and while many consumers are eager to adopt more sustainable eating habits, their preference for meat often presents a challenge. It is recommended to reduce meat consumption for environmental and health reasons (54), and blue mussels could serve as a sustainable substitute for meat (10). However, this provides that the umami flavor satisfies consumer preferences (13, 55). Flavor is a crucial factor when considering new foods for the future, making blue mussels a compelling option, for sustainability (11, 56), health, palatability and flavor.

### 4.2 Lipids and fatty acids

Utilizing the gold standard for lipid extraction, the Folch method yielded nearly twice the amount of lipids compared to the ethyl acetate method, as shown in Figure 3. This pattern was consistent across all three processing groups. Consequently, we used the lipid contents obtained from the Folch method for further analysis in this study and dismissed the ethyl acetate method as a potential lipid extraction method for blue mussels. Blue mussels should be considered a lean protein source as they contain low levels of lipids, 2.67 ± 0.11 g/100 g (Table 1). However, the fatty acid composition is highly beneficial. In the steamed mussels, approximately 50% of the FAs were PUFAs, and most of these PUFAs were EPA and DHA. Thus, blue mussels are an excellent source of LC-PUFA, particularly when compared to the increasingly consumed omega-6-rich, highly processed foods (57, 58). To use a nutritional claim that a food is high in omega-3 fatty acids, it must contain at least 80 mg EPA + DHA per 100 g and per 100 kcal (59). Consumption of 100 grams of steamed blue mussels will provide 271 mg EPA and 220 mg DHA, covering both the required intake of omega-3 for adults (250–500 mg EPA and DHA) and the recommendation of 200 mg DHA for pregnant and lactating women (36, 37).

### 4.3 Freeze-dried blue mussels as a potential feed ingredient for farmed Atlantic salmon

The nutrient contents of freeze-dried blue mussels in this study are particularly relevant when considering their use as fish feed ingredients. If used to feed farmed Atlantic salmon, the material would usually be processed into dry pellets, and both EAA and fatty acids were well preserved during freeze-drying (Tables 2, 5). The most prevalent amino acid was glutamic acid (comprised of both glutamine and glutamic acid in this analysis), 71.0 ± 2.71 mg/g DW in TAA. It has previously been demonstrated that the firmness of salmon filets was improved by supplementing the feed with glutamine and glutamic acid (60). In recent years, concerns have been raised about the declining proportion of omega-3 LC-PUFAs in farmed salmon fillets for human consumption, attributed to the decreased inclusion of marine ingredients in the salmon feed (21, 24). Comparing the contents of EAA in freeze-dried blue mussels to the requirements of Atlantic salmon (Table 4), blue mussels powder contained 9 out of the 10 EAA required in excessive quantities, covering 144–275% of the requirements. Although methionine is an exception, covering only 85% of the requirement, blue mussels should still be classified as an excellent source of this amino acid. It is also important to note that the content of the sulfur-containing amino acids methionine and cysteine together will meet the requirement of sulfur-containing amino acids, as already mentioned above.

After freeze-drying, the contents of LC-PUFA were found to be well preserved, with 10.4 ± 1.98 g EPA and 7.97 ± 1.49 g DHA per 100 g DW. Incorporating blue mussels into salmon feed could enhance the nutritional quality by increasing the omega-3 content (20). Furthermore, unlike vegetable sources, blue mussels do not contain indigestible fibers and less anti-nutrients (61), making them easier to digest and enhancing the bioavailability of the nutrients.

Health and welfare concerns are increasingly important, as Norwegian salmon farms are struggling with fish diseases and high mortality (62). Therefore, providing a diet that promotes both growth and health is of high priority. Given that blue mussels are a part of the natural diet for many marine species, they represent an attractive salmon feed ingredient, as they more closely mimic the natural diet compared to terrestrial vegetable sources. Recent studies have shown that incorporating blue mussel meal or blue mussel silage into salmon feed has been successful (27, 63). Additionally, feeding trials with mice fed salmon that included blue mussels in their diet reported no adverse effects on the mice (27, 64). Locally produced mussels could improve Norway’s self-sufficiency in aquafeed resources, contributing to increased food security and reducing the climate impact associated with transporting vegetable feed ingredients from other parts of the world (65). Incorporating blue mussels into salmon feed would address nutritional deficiencies and align with sustainable and health-conscious aquaculture practices (66).

### 4.4 Comparing the environmental footprints of farmed blue mussels and salmon

Several projects have tried to identify the nutrient benefits and possible challenges of utilizing different food alternatives in our diet (1, 67). Farmed food from the ocean will be needed as a source of food and nutrients in the future (2). When increasing the production to acquire more ocean food, it is necessary to balance the environmental impact of the production and the contribution of nutrients. Farmed blue mussels are non-fed organisms and comes with a lower environmental footprint than alternative farmed ocean food (68). The attempts to evaluate the environmental impact and the nutritional contribution of food alternatives have been increasing (69). Only a few footprints, such as global warming potential, eutrophication potential, and acidification potential, are frequently studied. Other footprints, such as the use of abiotic resources and water, are far less studied (56). As shown in this study, blue mussels are a good source of both EPA + DHA and EAA. In parallel, the recommended source of EPA and DHA are fatty fish like salmon, trout, marcel or herring (67), species also high in essential amino acids (70). The content of EPA + DHA in farmed salmon has decreased from 2005 to 2011 but has remained stable at around 1.0–1.3 g/100 g since then. The median protein content ranged from 18 to 20 g/100 g between 2005 and 2020 (20), both exceeding the contents in our mussels. The environmental footprint of different production methods (e.g., open/closed aquaculture on either land/sea) of farmed Atlantic salmon has been reviewed previously (71). The footprint has a broad range, depending on the production methods, and may potentially have a global warming potential of between 2,404 and 6,414 kg CO_2_ equivalents (eq.); acidification potentials of 15.1–26.7 kg SO_2_ eq.; and eutrophication potentials of 17.3 kg—26.7 PO_4_ eq. pr ton live weight of salmon. For farmed blue mussels these footprints ranged between 9.52–527 kg CO_2_ eq.; −0.89—0.44) kg PO_4_ eq.; and 0.3–6.5 kg SO_2_ eq. per ton, whole mussel (56). Assuming a 65% edible ratio in salmon and 15% for blue mussels (72, 73), it is possible to compare the environmental burdens and the access to nutrients. For both EPA + DHA and protein, blue mussel was found to provide these nutrients with either far less or very similar footprints compared to salmon, depending on the production system used. In addition, blue mussels may contribute beneficially to the biosequestration of oceanic carbon and nitrogen/phosphorus. The impact and the scale of the sequestration are still debated (74), but it may nonetheless potentially reduce the surplus of global warming gasses eutrophication nutrients (75). The environmental footprint might impact consumption advises in the future.

A strength of this study lies in the methodology of measuring the amino acids for determining the protein content of blue mussels, providing accurate and reliable results (40). Also, analyzing homogenate from 2 kg mussels instead of individual blue mussels removes intra-individual differences between the mussels. However, a limitation of this study is that the freeze-dried blue mussels were steamed, and analyses of raw, freeze-dried mussels are missing. Hence, future research should consider including freeze-dried raw blue mussels in the analyses. Exploring the direct effects of blue mussel consumption in more pre-clinical animal and human dietary intervention studies will be necessary. Examining the digestion and bioavailability of nutrients from blue mussels would provide valuable insights. One challenge of using mussels as a feed ingredient is the competition with human food resources, ethically as well as economically. Moreover, unlike more processed foods with longer shelf life, blue mussels, as a fresh product, present potential challenges with storage and distribution. In summary, while mussels offer promising nutritional benefits, further research and changes in food production and consumption practices are needed to fully leverage the potential.

## 5 Conclusion

This study underscores the nutritional advantage of blue mussels. And reveals that steaming and freeze-drying effectively preserve the AA and FA in blue mussels. A 100-gram serving of steamed blue mussels significantly meets the daily recommended EAA intake for humans and provides both EPA and DHA in substantial amounts. Compared to salmon, blue mussels offer these nutrients with lower or comparable environmental impact on global warming, eutrophication, and acidification, depending on the production system. Additionally, this study also demonstrates that the ethyl acetate method is ineffective for extracting lipids from blue mussels.

## Data Availability

The raw data supporting the conclusions of this article will be made available by the authors, without undue reservation.
