# Supplementary material for: Farmed blue mussels (Mytilus edulis)—a nutrient-dense resource retaining nutritional value through processing
Source: Front Nutr. 2024 Nov 1;11:1443229. doi: 10.3389/fnut.2024.1443229 (PMC11563823; doi:10.3389/fnut.2024.1443229)
Supplement: Supplementary file 1 [file Table_1.DOCX]

Supplementary Material

**Supplementary Table 1.** Proximate composition of blue mussels. All results are displayed as g/100 g wet weight (WW). Values are mean ± standard deviation of n=5 for water and ash content and n=6 for protein and lipids (Folch’s extraction). Within each column, statistical differences between processing groups are denoted with different letters.

| Sample | Water | Protein | Lipid | Ash | Carbohydrate  (Calculated) |
| --- | --- | --- | --- | --- | --- |
| Raw (WW) | 78.7 ± 0.21 ^a^ | 7.18 ± 0.25 ^a^ | 1.94 ± 0.20 ^a^ | 2.08 ± 0.03 ^a^ | 10.08 |
| Steamed (WW) | 78.6 ± 0.21 ^a^ | 9.29 ± 0.24 ^b^ | 2.67 ± 0.11 ^b^ | 1.54 ± 0.04 ^b^ | 7.88 |
| Steamed freeze-dried (WW) | 1.48 ± 0.06 ^b^ | 40.7 ± 1.18 ^c^ | 12.3 ± 0.18 ^c^ | 6.75 ± 0.09 ^c^ | 38.8 |
